# Supplementary material for: Characterizing the Alteration in Rumen Microbiome and Carbohydrate-Active Enzymes Profile with Forage of Muskoxen Rumen through Comparative Metatranscriptomics
Source: Microorganisms. 2021 Dec 30;10(1):71. doi: 10.3390/microorganisms10010071 (PMC8777777; doi:10.3390/microorganisms10010071)
Supplement: Supplementary file 1 [file microorganisms-10-00071-s001.zip › microorganisms-1500983-supplementary.pdf]

Table S1. Comparison of chemical composition between triticale straw and brome hay (% dry matter)

Component, %	Triticale straw	Brome hay	*P* value
Dry Matter	87.8 ± 3.91	87.5 ± 2.65	0.220
Neutral Detergent Fiber	83.7 ± 3.49*	69.9 ± 0.29	0.002
Acid Detergent Fiber	52.2 ± 3.66*	37.8 ± 0.31	0.003
Cellulose	43.7 ± 2.73*	32.7 ± 0.51	0.013
Hemicellulose	31.8 ± 1.63	31.8 ± 1.63	0.170
Lignin	7.4 ± 1.30	4.9 ± 0.82	0.090
Nitrogen	0.67 ± 0.11*	1.12 ± 0.10	0.001

Table S2. Summary of sequencing output

Feeds	Animals	Sample ID	Total reads	LSU		SSU	
				Hit Reads number	% of total reads	Hit Reads number	% of total reads
1st triticale straw	Odin	777	240,614	119,883	49.82	120,725	50.17
	Carter	789	279,030	138,232	49.54	140,797	50.46
	Yessey	783	1,786,228	1,059,874	59.34	725,136	40.60
	Willie	797	375,027	188,342	50.22	186,683	49.78
2nd brome hay	Odin	1,014	1,047,269	560,762	53.55	485,745	46.38
	Carter	1,004	85,868	41,679	48.54	44,003	51.24
	Yessey	1,023	637,292	275,425	43.22	360,897	56.63
	Willie	1,021	879,880	493,437	43.82	385,528	56.08
1st brome hay	Bernie	771	875,018	495,986	56.68	378,312	43.23
	Kobuk	791	1,241,700	501,858	40.42	737,229	59.37
	Tyr	780	1,899,935	1,005,533	52.92	893,413	47.02
2nd triticale straw	Bernie	1,001	60,949	29,462	48.34	31,483	51.65
	Kobuk	1,008	366,827	178,812	48.75	188,011	51.25
	Tyr	1,018	1,268,690	649,566	51.20	619,122	48.80

LSU: Large Subunit rRNA; SSU: Small Subunit rRNA.

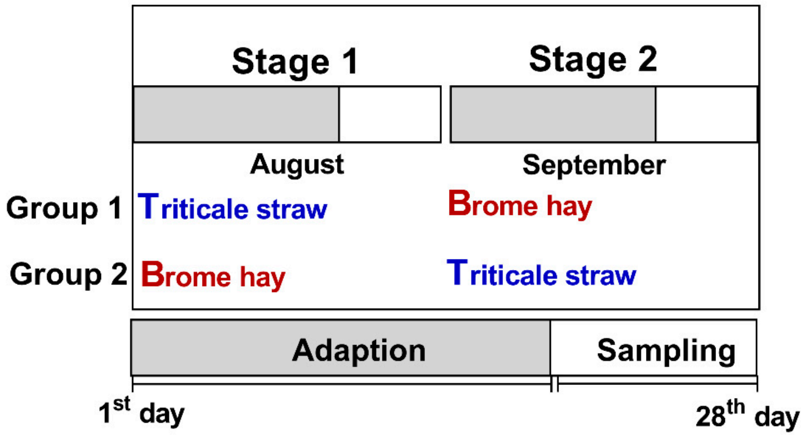

Figure S1 Schematic presentation of a crossover design, 8 castrated muskoxen with ruminal cannulas were housed in individual pens and assigned into two groups based on their initial body mass (268 ± 18.4 kg vs. 278 ± 27.2 kg), and ad libitum fed with triticale (*Triticosecale hexaploide*) straw as a low-quality diet or brome (*Bromus spp*.) hay as a medium-quality diet from August to September
